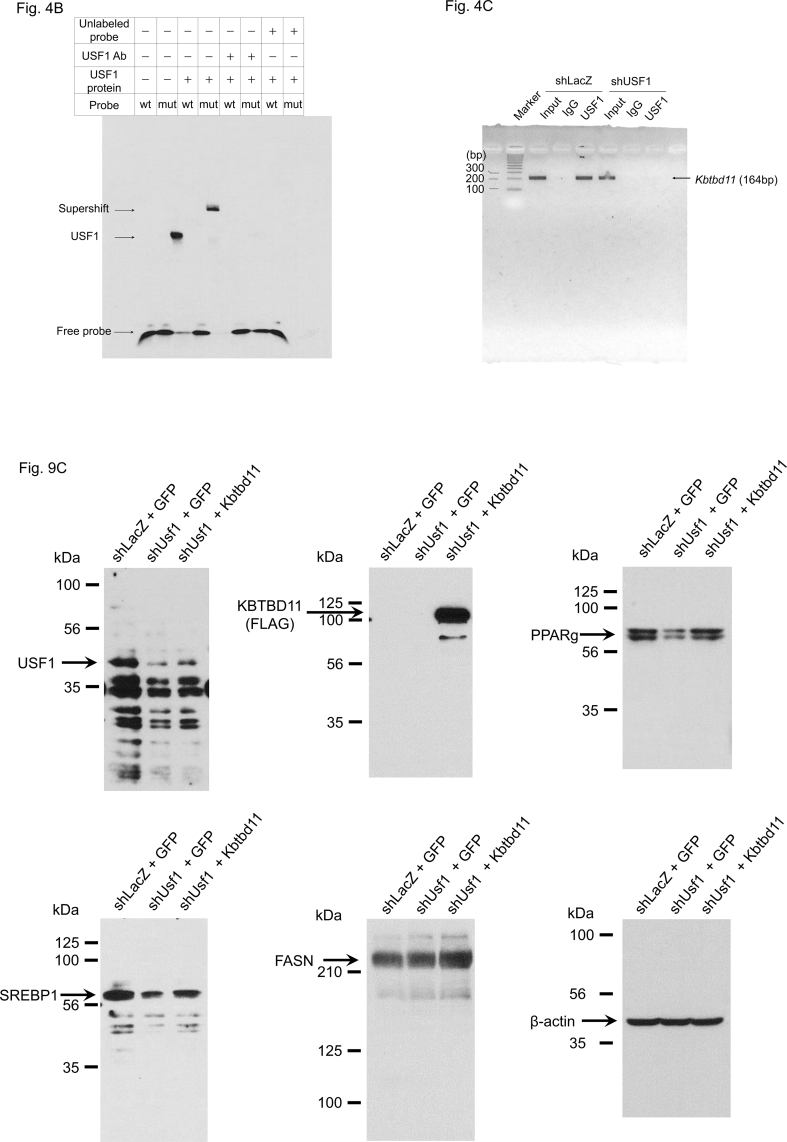# *Kbtbd11* contributes to adipocyte homeostasis through the activation of upstream stimulatory factor 1

**DOI:** 10.1016/j.heliyon.2019.e02777

**Published:** 2019-11-14

**Authors:** Kazuhisa Watanabe, Kazuha Yokota, Ken Yoshida, Ayumi Matsumoto, Sadahiko Iwamoto

**Affiliations:** Division of Human Genetics, Center for Molecular Medicine, Jichi Medical University, 3311-1 Yakushiji, Shimotsuke, Tochigi, 329-0498, Japan

**Keywords:** Cell biology, Cell differentiation, Gene expression, Gene regulation, Transcription factor, Biochemistry, Molecular biology, Obesity, *Kbtbd11*, 3T3-L1 differentiation, USF1, Transcriptional regulation

## Abstract

The present study aimed to investigate the transcriptional regulation of *Kbtbd11* in adipose tissue. To elucidate the physiological role of *Kbtbd11* gene expression, adipose *Kbtbd11* mRNA expression levels were estimated under various feeding states in wild-type mice. *Kbtbd11* expression increased in a time-dependent manner in the adipose tissue in mice fed on chow diet, whereas the promotion of *Kbtbd11* mRNA expression by refeeding was attenuated in mice fed on high-fat (HF) diet, suggesting the suppression of *Kbtbd11* mRNA expression under HF diets and that changes in mRNA levels were associated with regulation of the transcription activity of *Kbtbd11* by some transcription factors. To investigate the transcriptional regulation of *Kbtbd11*, the fragment upstream of either mouse *Kbtbd11* or human *KBTBD11* promoter was inserted into a luciferase vector. Luciferase reporter assays revealed that both mouse and human *KBTBD11* promoter activity was increased by USF1. Direct USF1 binding to the Ebox in the *Kbtbd11* promoter was confirmed by electrophoretic mobility shift and chromatin immunoprecipitation assays. In addition, the adipocyte differentiation marker levels increased instantly in *Kbtbd11*-overexpressing *Usf1* knockdown cells than in *Usf1* knockdown cells. These results imply an association of between *Kbtbd11* with *Usf1* expression and suggest the involvement of *Kbtbd11* in a novel adipogenesis pathway.

## 1 Introduction

Kelch repeat and BTB domain-containing 11 (KBTBD11) is a member of the *KBTBD* subfamily, which comprised BTB/POZ and Kelch domains. The BTB/POZ domain functions as the protein–protein interaction domain to facilitate dimer formation and interaction with nonBTB domain comprising proteins, involving activities such as transcriptional regulation, cytoskeleton dynamics, ion channel assembly and gating, and protein ubiquitination/degradation [1]. The Kelch domain, which is widely conserved in mammals and insects, usually comprises 2–7 repeats of four-stranded beta-sheet motifs that form the beta-propeller structure [2]. The Kelch β-propellers primarily function as scaffolds for protein–protein interactions. Kelch proteins interact directly with actin, suggesting the regulation of cell–cell interactions, cell–substrate interactions, and cell migration [3].

The putative tumor suppressor gene *KBTBD11* is regulated by MYC. A variant allele of *KBTBD11*—rs11777210—is significantly associated with cell susceptibility to colorectal cancer. *KBTBD11* expression is significantly decreased in tumor tissues compared with adjacent paired normal tissues [4]. We have previously reported that *Kbtbd11* is involved in nutritional regulation and is highly expressed in the epididymal white adipose tissue (eWAT) in diet-induced obesity (DIO) mice compared with that in mice fed on chow diet [2]. In addition, the adenovirus-mediated knockdown of *Kbtbd11* in 3T3-L1 cells inhibits mitotic clonal expansion (MCE), which is required during the early stages of 3T3-L1 adipocyte differentiation [2]. In contrast, *Kbtbd11*-overexpressing 3T3-L1 promotes MCE, which leads to the expression of adipocyte differentiation markers*—C/ebpa* and *Pparg*, and induces lipid accumulation [2], suggesting that *Kbtbd11* expression levels play a major role in MCE and influence triglyceride accumulation and adipocyte differentiation. In this context, the present study aimed to clarify the transcriptional regulation of *Kbtbd11* to elucidate the functions underlying the role of *Kbtbd11* in adipocyte differentiation.

## 2 Materials and methods

### 2.1 Animal experiments

For mice experiments, we used 8-week-old male C57BL/6 mice from CLEA Japan. The mice were maintained on a normal chow diet. For fasting–refeeding experiments, C57BL/6 mice were fasted for 24 h and then fed a chow diet or HF diet for 4, 6, 8 and 12 h. Feed ingredient contents were as follows: normal chow diet (CE-2) comprised carbohydrate 50.3%, protein 25.4%, and fat 4.4% and high-fat diet (HFD32) comprised carbohydrate 29.4%, protein 25.5%, and fat 32.0% (CLEA Japan Inc.). Animal experimental protocols were approved by the Animal ethics committee of Jichi Medical University (permit number 17177).

### 2.2 Cell and adipocyte differentiation

**Fig. 1:**
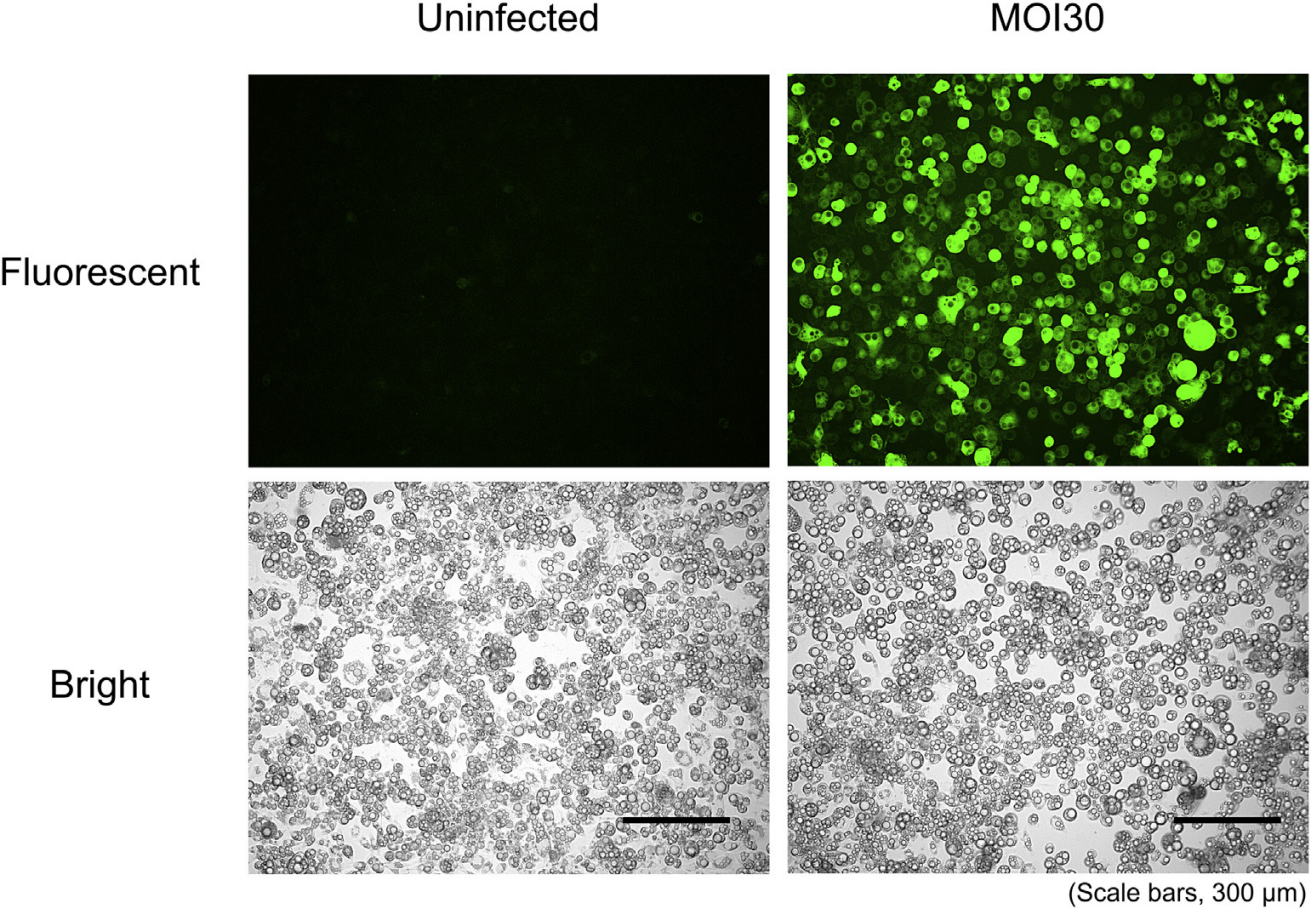
The expression of GFP in 3T3-L1 cells. Mature 3T3-L1 cells were infected with Ad-CMV-GFP at an MOI of 30; after 48 h, the cells were observed to fluoresce (scale bar = 300 μm).

Human kidney 293T (HEK293T) and 3T3-L1 cells were maintained in low-glucose Dulbecco's modified Eagle's medium (DMEM) supplemented with 10% fetal bovine serum and 100 units each of penicillin and streptomycin at 37°C in 5% CO_2_. For adipocyte differentiation experiments, at 2 days after 3T3-L1 cells confluence, the medium was replaced with high-glucose DMEM comprising insulin (5 μg/mL), dexamethasone (1 μM), and 3-isobutyl-1-methylxanthine (0.5 mM). After 2 days of incubation, the medium was replaced with high-glucose DMEM comprising only 5 μg/mL insulin. The medium was replaced every alternate day. For adenovirus infection experiments, 3T3-L1 cells at day −2 after induction of differentiation were infected with adenovirus. 3T3-L1 cells were infected with the adenovirus at a multiplicity of infection (MOI) of 10 plaque-forming units per cell. The adenovirus used in this study demonstrated to be expressed with an efficacy of almost 100% at an MOI of 30 in 3T3-L1 adipocytes, as assessed by GFP (Fig. 1).

### 2.3 Oil Red O (ORO) stain

Adipocytes were fixed with 10% formalin solution in phosphate-buffered saline (PBS) for 10 min and replaced with 60% isopropanol in PBS for 1 min. The fixed adipocytes were stained with ORO for 20 min at room temperature.

### 2.4 Transfection and luciferase assays

The Dual-Luciferase Reporter Assay System (Promega) was used to determine promoter expression levels. Mouse *Kbtbd11* and human *KBTBD11* were generated using PCR with either mouse or human genomic DNA as a template. 3T3-L1 cells were co-transfected with each expression vector, mouse *Kbtbd11* and human *KBTBD11* promoters that drive firefly luciferase expression (pGL4.10 *mKbtbd11*-Luc and *hKBTBD11*Luc) and *Renilla reniformis* luciferase vector (pGL4.74) for use as an internal control reporter. The cells were incubated for 24 h post-transfection at 37°C in 5% CO_2_ and lysed in 100 μL 1x Passive Lysis Buffer (Promega). Lysate was used for the luciferase assay, and luminescence was detected using a Luminometer (Thermo Fisher Scientific).

### 2.5 Electrophoretic mobility shift assay (EMSA)

The probes shown in Fig. 4A were synthesized by Thermo Scientific and biotin labeled. HEK293T cells were transfected with a USF1 expression vector driven by a CMV promoter. Nuclear protein was extracted 48 h post-transfection. The biotin-labeled probe was mixed with the USF1-overexpressing nuclear protein extract [super-shift lane was generated by adding the USF1 protein and USF1 antibody (sc-229, SantaCruz Biotech)] and allowed to incubate at room temperature for 20 min. For detection of the DNA-protein bands, we used the LightShift Chemiluminescent EMSA kit (Thermo Fisher Scientific). The wild-type (wt) and mutant (mut) probes for EMSA were as follows: *Kbtbd11* Ebox (wt), TTTTCTCCACCCACGTGTAAATG and *Kbtbd11* Ebox (mut), TTTTCTCCACCCAaaaGTAAATG.

### 2.6 Chromatin immunoprecipitation (ChIP) assay

For the ChIP assay, we used the SimpleChIP Enzymatic Chromatin IP Kit (Cell Signaling Technology). Immunoprecipitation was performed using an USF1 tag antibody with mouse IgG as the negative control. After immunoprecipitation, the associated DNA was amplified with a primer pair: *Kbtbd11* promoter−832 Fwd 5′-CCGCATCCTGGTCACCTTTC-3′ and *Kbtbd11* promoter−669 Rv 5′- CCTTCCTTCCGTTCCTGTTGG-3′.

### 2.7 Adenoviral expression vectors

For adenoviruses amplification, we used ViraPower Adenoviral Expression System (Thermo Fisher Scientific) as described previously [5]. Full-length *Kbtbd11*-cDNA was subcloned into the pENTR/D-TOPO vector (Thermo Fisher Scientific). The pENTR-*Kbtbd11* (C-terminal FLAG-tagged) vector was transferred into the pAd/CMV/V5-DEST vector using the Gateway system (Thermo Fisher Scientific). Sequences corresponding to the shRNAs for *Kbtbd11* and *lacZ* were cloned into pBlock-it (Thermo Fisher Scientific). The sequence of the shRNA for *Usf1*: 5′-cacc GTACGTCTTCCGAACTGAG acgtgtgctgtccgt CTCAGTTCGGAAGACGTAC-3′. The adenoviruses were purified using ViraBind Adenovirus Miniprep Kit (Cell Biolabs) according to the manufacturer's protocol.

### 2.8 Real-time PCR (RT-PCR)

Total RNA was extracted with acid guanidinium thiocyanate–phenol reagents [6]. cDNA synthesis was performed using 1 μg of total RNA each and the Verso cDNA Kit (Thermo Fisher Scientific) with random hexamer primers. RT-PCR assays were performed using the ViiA7 Real-Time PCR System and Kapa SYBR fast universal qPCR kit (Kapa Biosystems). The following primers were used for this analysis: *Kbtbd11* Fwd, 5′-TCAGCGTTTTCCGCTACCAT-3′ and *Kbtbd11* Rv, 5′-AACACAACGAAAGGGCTGGA-3′; *C/ebpa* Fwd, 5′-GCCATGCCGGGAGAACTCTA-3′ and *C/ebpa* Rv, 5′-GGGCTCTGGAGGTGACTGCT-3′; *Pparg* Fwd, 5′-TTCCACTATGGAGTTCATGCTTGT-3′ and *Pparg* Rv, 5′-TCCGGCAGTTAAGATCACACCTA-3′; *Usf1* Fwd, 5′-ACCCTTATTCCCCGAAGTCAGA-3′ and *Usf1* Rv, 5′-CGGCGCTCCACTTCGTTATGT-3′; *Srebp1c* Fwd, 5′-CGGCGCGGAAGCTGT-3′ and *Srebp1c* Rv, 5′-TGCAATCCATGGCTCCGT-3′; *Fasn* Fwd, 5′-ATCCTGGAACGAGAACACGATCT-3′ and *Fasn* Rv, 5′-AGAGACGTGTCACTCCTGGACTT-3′; *aP2* Fwd, 5′-TTTCCTTCAAACTGGGCGTG-3′ and *aP2* Rv, 5′-AGGGTTATGATGCTCTTCACCTTC-3′; *Tnfα* Fwd, 5′-CAGCCGATGGGTTGTACCTT-3′ and *Tnfα* Rv, 5′-GGGCTCATACCAGGGTTTGA-3′; *Il6* Fwd, 5′-GAGGATACCACTCCCAACAGACC-3′ and *Il6* Rv, 5′-AAGTGCATCATCGTTGTTCATACA-3′; *Bax* Fwd, 5′-GCTGACATGTTTGCTGATGG-3′ and *Bax* Rv, 5′-GATCAGCTCGGGCACTTTAG-3′; *Bcl2* Fwd, 5′-CTGGGATGCCTTTGTGGAAC-3′ and *Bcl2* Rv, 5′-GAGACAGCCAGGAGAAATCAAAC-3′; *Rplp0* Fwd, 5′-ATGCAGCAGATCCGCATGT-3′ and *Rplp0* Rv, 5′-TTGCGCATCATGGTGTTCTT-3′.

### 2.9 Western blotting

Whole cell lysates were electrophoretically separated on denaturing polyacrylamide gels and transferred to polyvinylidene difluoride membranes. Proteins were detected with USF1 (sc-229, Santa Cruz Biotechnology), FLAG (F1804, Merck), PPARg (2443, Cell Signaling Technology), SREBP1 (sc-365514, Santa Cruz Biotechnology), FASN (3180 Cell Signaling Technology), and β-actin antibody (G043, abm).

### 2.10 Statistical analysis

Statistical significance was tested using the unpaired two-tailed Student's *t*-test. All data were expressed as mean ± SEM. Statistical significance was set at *p* < 0.05.

## 3 Results

### 3.1 *Kbtbd11* expression in epididymal WAT (eWAT) after feeding

**Fig. 2:**
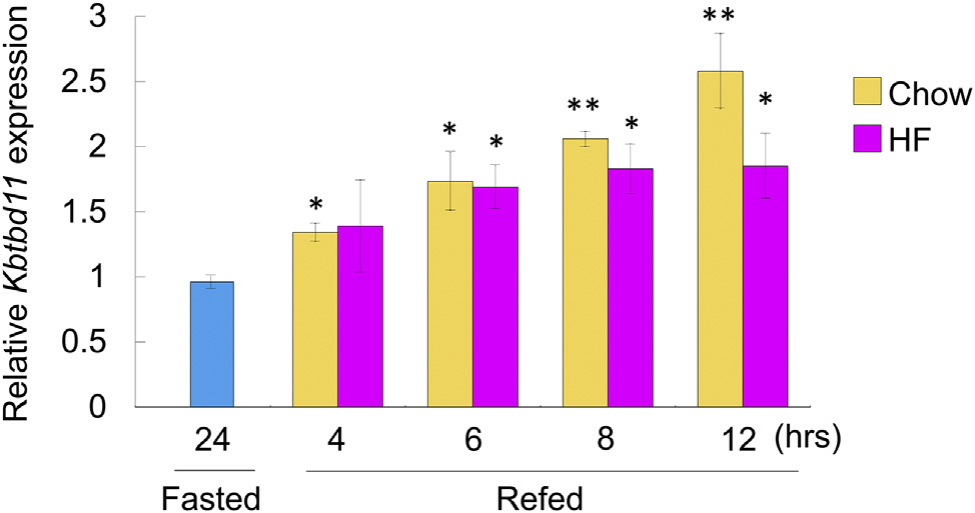
Epididymal white adipose tissue (eWAT) mRNA expression levels of *Kbtbd11* under various feeding states. The expression of *Kbtbd11* in the eWAT of wild-type mice: Mice were fasted for 24 h or fasted for 24 h/refed for 4, 6, 8, and 12 h. Wild-type mice were fed on chow diet or HF diet during the refeeding period. *n* = 3 per group, *p < 0.05, **p < 0.01 vs. fasted.

We have previously reported that *Kbtbd11* expression is upregulated at 12 h after a chow diet feeding and is increased in the adipose tissue in HF DIO mice [2]. To elucidate the nutritional regulation of *Kbtbd11* expression in eWAT at an earlier time after feeding, the gene expression levels under various feeding states were estimated in C57BL6/J wild-type mice. *Kbtbd11* mRNA expression was increased in a time-dependent manner during chow die refeeding. The increase in *Kbtbd11* expression after feeding was attenuated in mice fed on HF diet compared with mice fed on chow diet (Fig. 2).

### 3.2 Regulation of *Kbtbd11* promoter by USF1

**Fig. 3:**
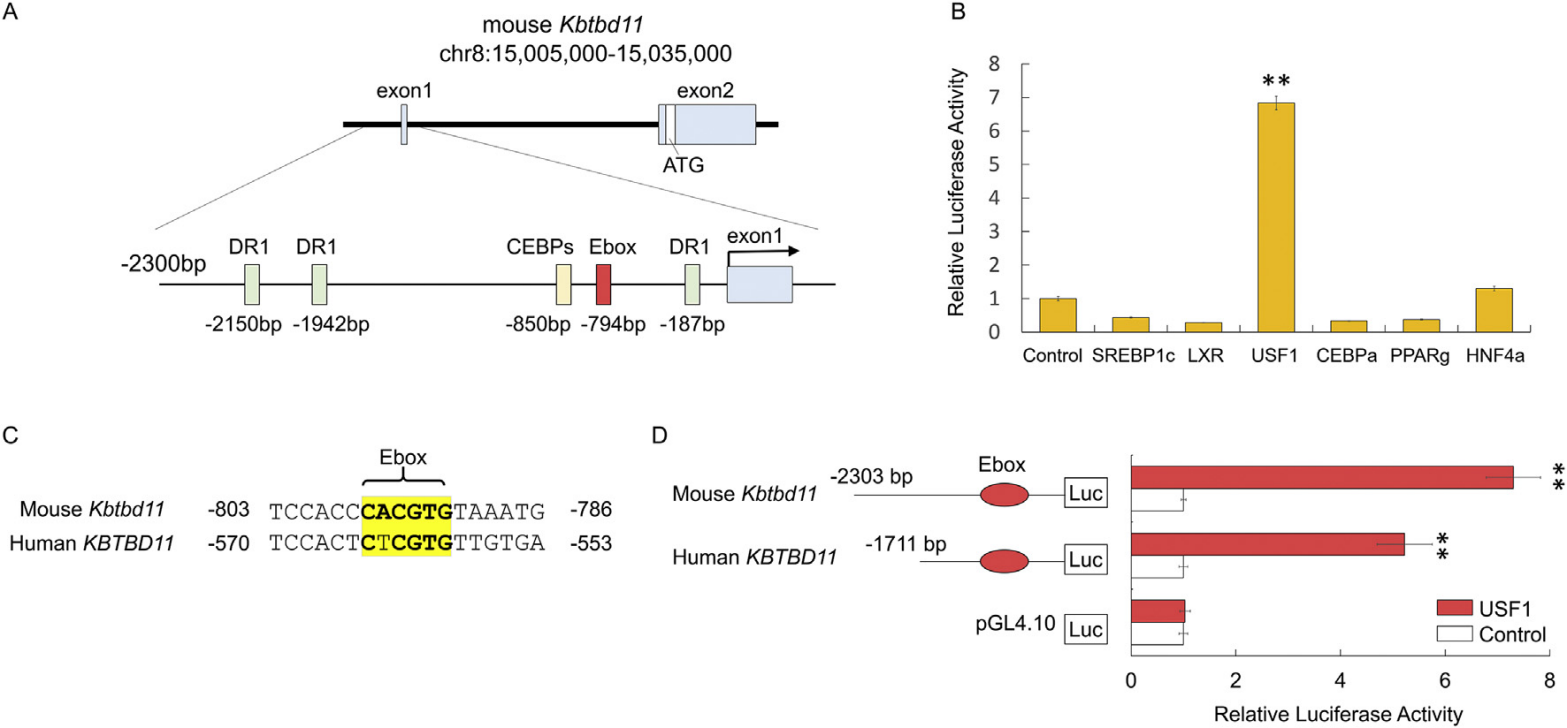
Activation of *Kbtbd11* promoter by USF1. (A) Schematic representation of mouse *Kbtbd11* promoter with the potential binding sites of lipid metabolism-related transcription factors. (B) The mouse *Kbtbd11* promoter region (2303 bp) was fused to a luciferase reporter gene (pGL4.10 *mKbtbd11*-2030-Luc). 3T3-L1 cells were cotransfected with pGL4.10 *Kbtbd11*-2303-Luc as the reporter gene, *R. reniformis* luciferase vector (pGL4.74) as the internal control reporter, and the indicated expression plasmids or control vector (pcDNA3.1). (C) Ebox of the mouse *Kbtbd11* and human *KBTBD11* promoters. (D) Effects of USF1 on the mouse *Kbtbd11* and human *KBTBD11* promoter activity in 3T3-L1 cells. *n* = 3 per group, **p < 0.01 vs. control.

The gene structure of mouse *Kbtbd11* is presented in Fig. 3A. To identify the potential binding sites of lipogenic and adipogenic transcription factors, we used LASAGNA-Search 2.0 [7]*.* We found putative transcription factor binding sites, such as direct repeat 1 (DR1), Ebox, and CCAAT/enhancer-binding proteins (C/EBPs) response elements. Sterol regulatory element-binding proteins (SREBPs), which are major transcriptional regulators of lipid metabolism, bind to the sterol regulatory element DNA sequence TCACNCCAC [8]. The lipid-activated transcription factors—liver X receptors (LXRs) and peroxisome proliferator-activated receptors (PPARs)—bind to the direct repeat motifs of the AGGTCA sequence separated by one nucleotide (DR1) [9]. Hepatocyte nuclear factor-4 (HNF4), which induces numerous genes involved in lipid metabolism, also binds to DR1 [10].

To determine whether such transcription factors activate the *Kbtbd11* promoter, the 2.3-kb 5′ region upstream of the mouse *Kbtbd11* promoter was cloned into a luciferase vector and a luciferase reporter assay was performed in 3T3-L1 cells. The *Kbtbd11* promoter was markedly activated by the co-expression of USF1 (Fig. 3B). In addition, we examined whether the Ebox motif was present in human *KBTBD11* promoter and found the predicted Ebox motif (5′-CTCGTG-3′) in the 0.5-kb 5′ region upstream of the human *KBTBD11* (Fig. 3C). The 1.7-kb 5′ region upstream of the human *Kbtbd11* promoter was cloned into a luciferase vector. The human *KBTBD11* promoter was also significantly activated by the co-expression of USF1 (Fig. 3D).

### 3.3 Ebox regions for USF1 activation of *Kbtbd11* promoter

**Fig. 4:**
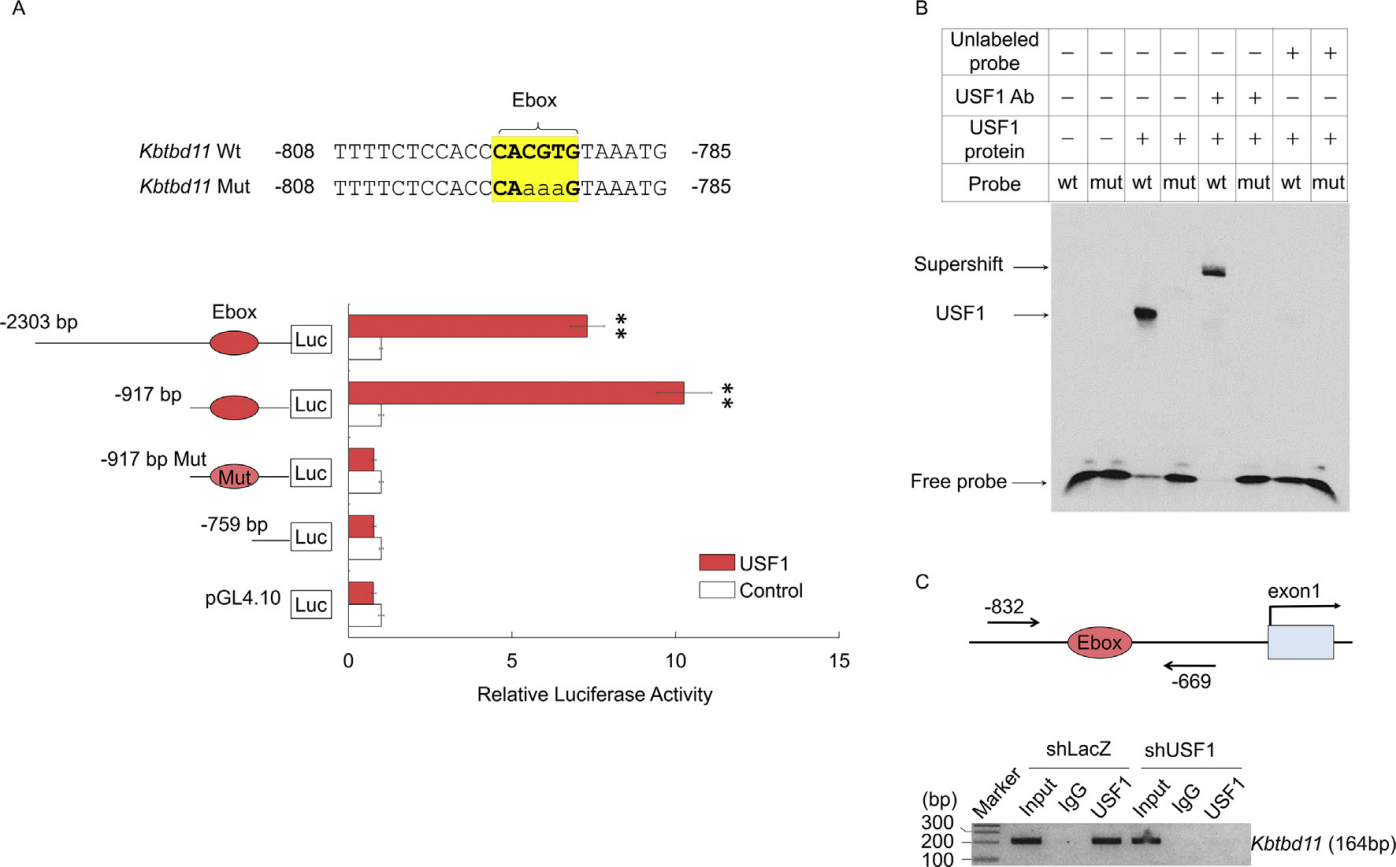
*Kbtbd11* promoter is a direct target of USF1. (A) Ebox of the mouse *Kbtbd11* and mutant *Kbtbd11* promoters. (B) Luciferase assays of the reporter gene pGL4.10 Kbtbd11-2303-Luc and its various deletion constructs in the presence or absence of the USF1 expression plasmid in 3T3-L1 cells. *n* = 3 per group, **p < 0.01 vs. control. (C) EMSA for USF1 binding to Ebox of *Kbtbd11* promoter. Nuclear extracts of USF1 protein were incubated with biotin-labeled *Kbtbd11* promoter or mutant *Kbtbd11* promoter probe in the presence or absence of unlabeled probes and USF1 antibody. The competition was performed using unlabeled probes as competitors in 1000-fold molar excess. (D) ChIP assay for USF1 binding to Ebox of the *Kbtbd11* promoter using infection of either shLacZ or shUsf1 knockdown adenoviruses in mature adipocytes after 3T3-L1 differentiation at day 8. The cells were harvested at day 2 post-infection. The extracted genomic DNA was subjected to immunoprecipitation, performed using and antibody against USF1, with IgG as a negative control. For comparison, amplification derived from unprecipitated chromatin is also shown (input).

To identify the Ebox sites of the *Kbtbd11* promoter for USF1 binding, various deletion and mutation constructs, as indicated in Fig. 4A, were prepared and evaluated according to the basal and USF1-induced activities. The region responsible for USF1 activation was located within −917 bp, indicating that the Ebox sequences at −796 and −791 bp were crucial for USF1 activation (Fig. 4A). To confirm the direct binding of USF1 to the Ebox sequence, we performed EMSA using wt and mut probes (Fig. 4A). To destroy the USF1 binding site in EMSA, the wt oligonucleotide 20-mer was base-substituted at three positions within the Ebox (Fig. 4A). The EMSA shows that USF1 binds only to the wt oligonucleotide and is super-shifted in the presence of the USF1 antibody (Fig. 4B). ChIP assay of genomic DNA from adenovirus-mediated knockdown of *Usf1* in 3T3-L1 adipocytes at day 8 of differentiation confirmed the direct binding of USF1 to the Ebox in vitro; however, the intensity signal detected at shLacZ was completely eliminated following USF1 knockdown (Fig. 4C). The results indicated that USF1 activated *Kbtbd11* gene transcription by binding specifically to the Ebox sequence in the *Kbtbd11* promoter.

### 3.4 *Usf1* expression in epididymal WAT (eWAT) after feeding

**Fig. 5:**
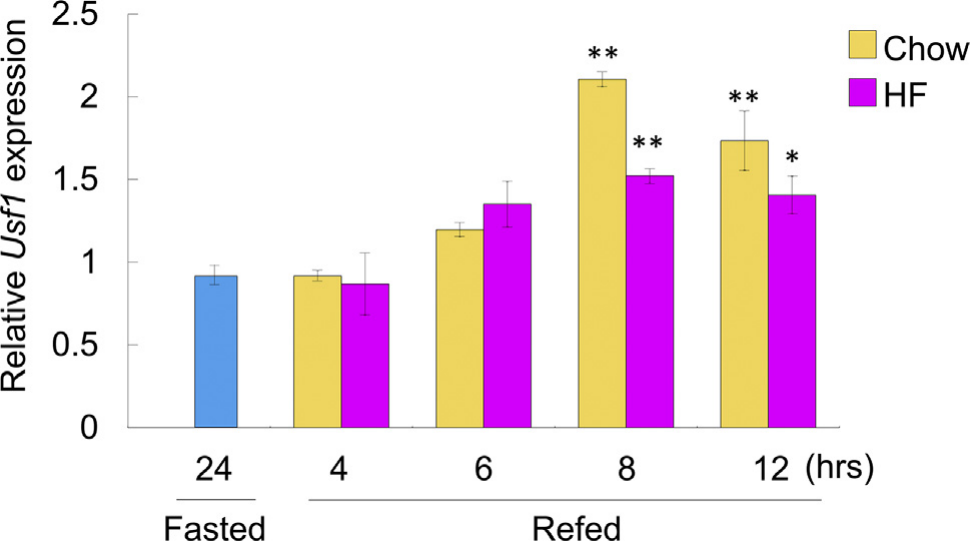
Epididymal white adipose tissue (eWAT) mRNA expression levels of *Usf1* in various feeding states. The expression of *Usf1* in the eWAT of wild-type mice: Mice were fasted for 24 h or fasted for 24 h/refed for 4, 6, 8, and 12 h. Wild-type mice were fed on chow diet or HF diet during the refeeding period. *n* = 3 per group, *p < 0.05, **p < 0.01 vs. fasted.

To elucidate the nutritional regulation of *Usf1* expression in eWAT after feeding, the gene expression levels were estimated under various feeding states in C57BL6/J wild-type mice. The *Usf1* mRNA expression was increased in a time-dependent manner during chow diet refeeding. The increase in *Usf1* expression after feeding was attenuated in mice fed on HF diet compared with that in mice fed on chow diet (Fig. 5).

### 3.5 Plasma insulin and glucose levels after feeding

**Fig. 6:**
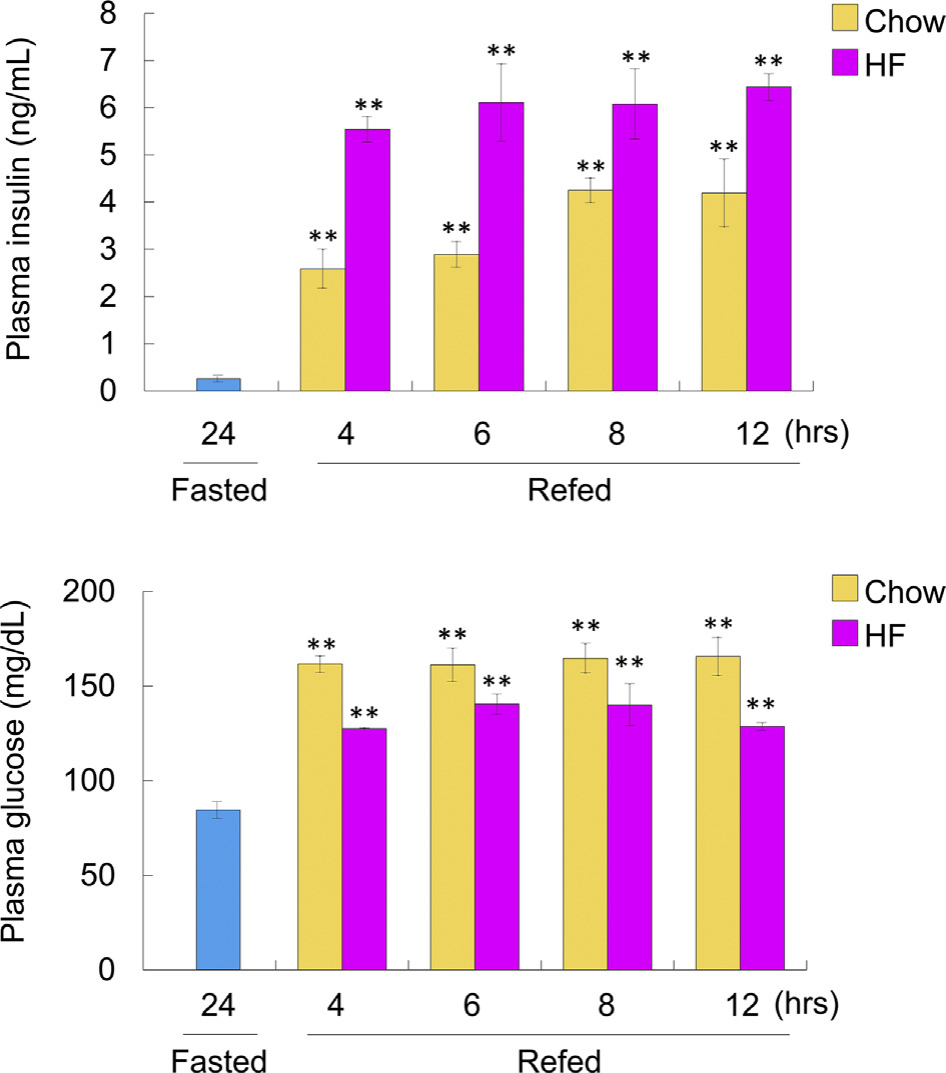
Plasma insulin and glucose levels under various feeding states. Upper panel, plasma insulin levels; bottom panel, plasma glucose levels: Mice were fasted for 24 h or fasted for 24 h/refed for 4, 6, 8, and 12 h. Wild-type mice were fed on chow diet or HF diet during the refeeding period. *n* = 3 per group, *p < 0.05, **p < 0.01 vs. fasted.

Because glucose increases *USF1* expression [11], *Kbtbd11* and *Usf1* expression levels might be affected by circulating insulin and/or glucose levels. To examine circulating insulin and glucose levels, plasma insulin and glucose levels were determined under various feeding states in C57BL6/J wild-type mice. Plasma insulin and glucose levels were significantly increased after refeeding. Plasma insulin levels were significantly increased during HF refeeding compared with that during chow diet refeeding, whereas plasma glucose levels were significantly decreased and plasma insulin levels were increased after HF refeeding (Fig. 6). These results suggested that *Kbtbd11* expression was affected by USF1 expression levels via insulin and glucose levels.

### 3.6 Effects of *Kbtbd11* overexpression on mature 3T3-L1 adipocytes

**Fig. 7:**
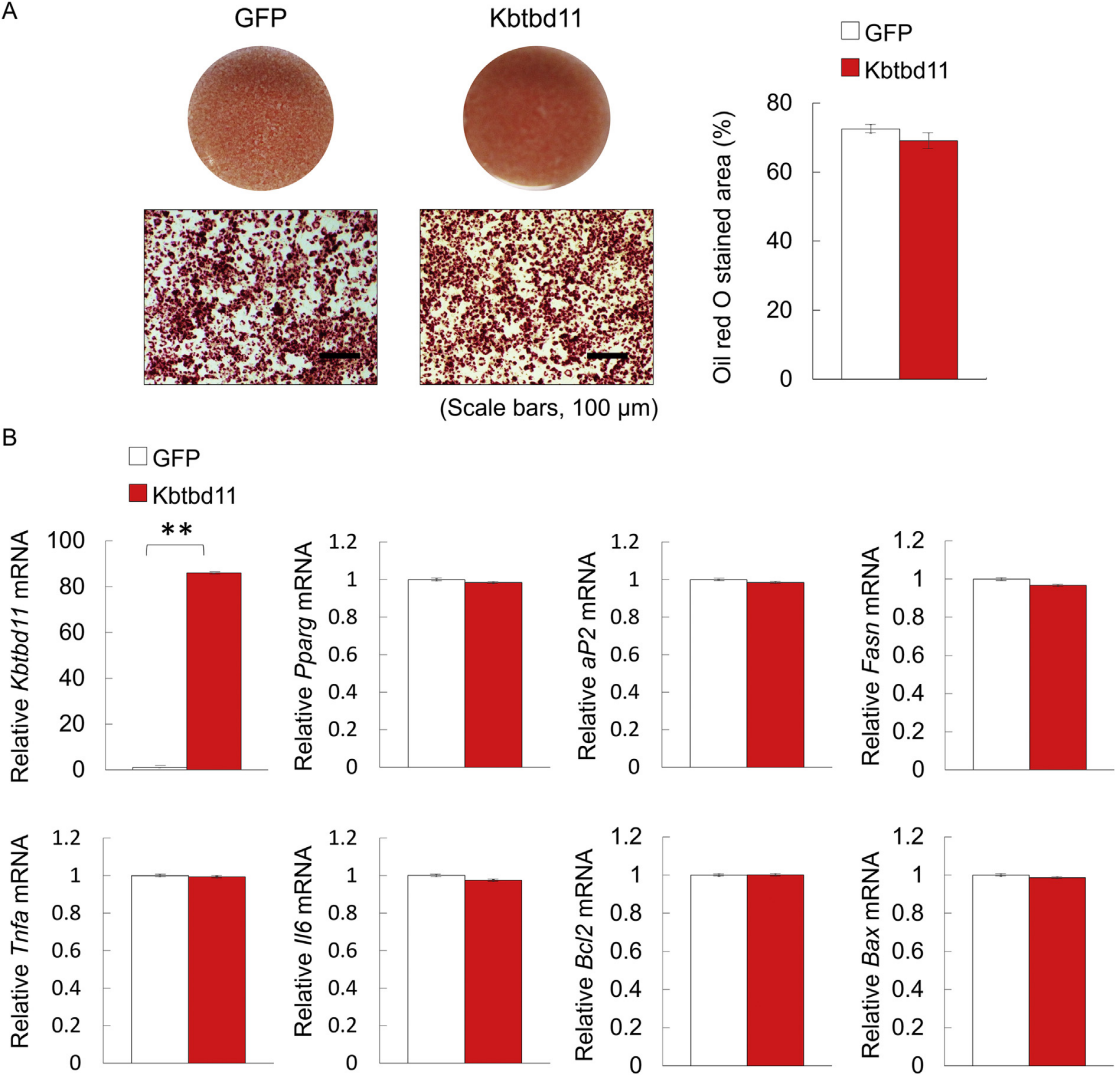
The effects of *Kbtbd11* overexpression on mature 3T3-L1 adipocytes. Mature 3T3-L1 cells were infected with adenoviral vectors (GFP and *Kbtbd11*) at an MOI of 30. (A) Triglyceride accumulation in *Kbtbd11* overexpression mature 3T3-L1 adipocytes at 48 h visualized using Oil Red O staining (scale bar = 100 μm) (left), quantification of Oil Red O stained area (right); (B) Total RNA was harvested at 48 h after adenoviral infection, and expression levels of *Kbtbd11*, adipogenic (*Pparg* and *aP2*) and inflammation markers (*Tnfα* and *Il6*), and lipogenic (*Fasn*) and proapoptotic genes (*Bax* and *Bcl2*) were measured using qPCR; *n* = 3 per group, **p < 0.01 vs. GFP.

Based on the changes in *Kbtbd11* expression levels in the livers of feeding mice, we were investigated the potential role of *Kbtbd11* in mature 3T3-L1 adipocytes by investigating the effects of *Kbtbd11* overexpression on mature 3T3-L1 adipocytes. At day 8 after inducing differentiation, mature 3T3-L1 adipocytes were infected with GFP control or *Kbtbd11* overexpression adenovirus. Compared with the GFP control, *Kbtbd11* overexpression increased the *Kbtbd11* expression levels by 80-folds (Fig. 7B). However, lipid droplet abundance, as observed with ORO staining of mature 3T3-L1 adipocytes, was not different between conditions under the GFP control and *Kbtbd11* overexpression (Fig. 7A). Consistent with the observations under ORO staining, the mRNA expression analyses of adipogenic (*Pparg* and *aP2*) and inflammation markers (*Tnfα* and *Il6*), in addition to lipogenic (*Fasn*) and proapoptotic genes (*Bax* and *Bcl2*), were not altered. We have previously demonstrated that the effects of *Kbtbd11* knockdown on mature 3T3-L1 adipocytes do not vary between the control and Kbtbd11 knockdown conditions [2]. Taken together, the results suggested that *Kbtbd11* plays a role after starvation and/or during lipid accumulation in adipose tissue.

### 3.7 Inhibition of adipocyte differentiation in *Usf1* knockdown 3T3-L1 cells was rescued by *Kbtbd11* overexpression

**Fig. 8:**
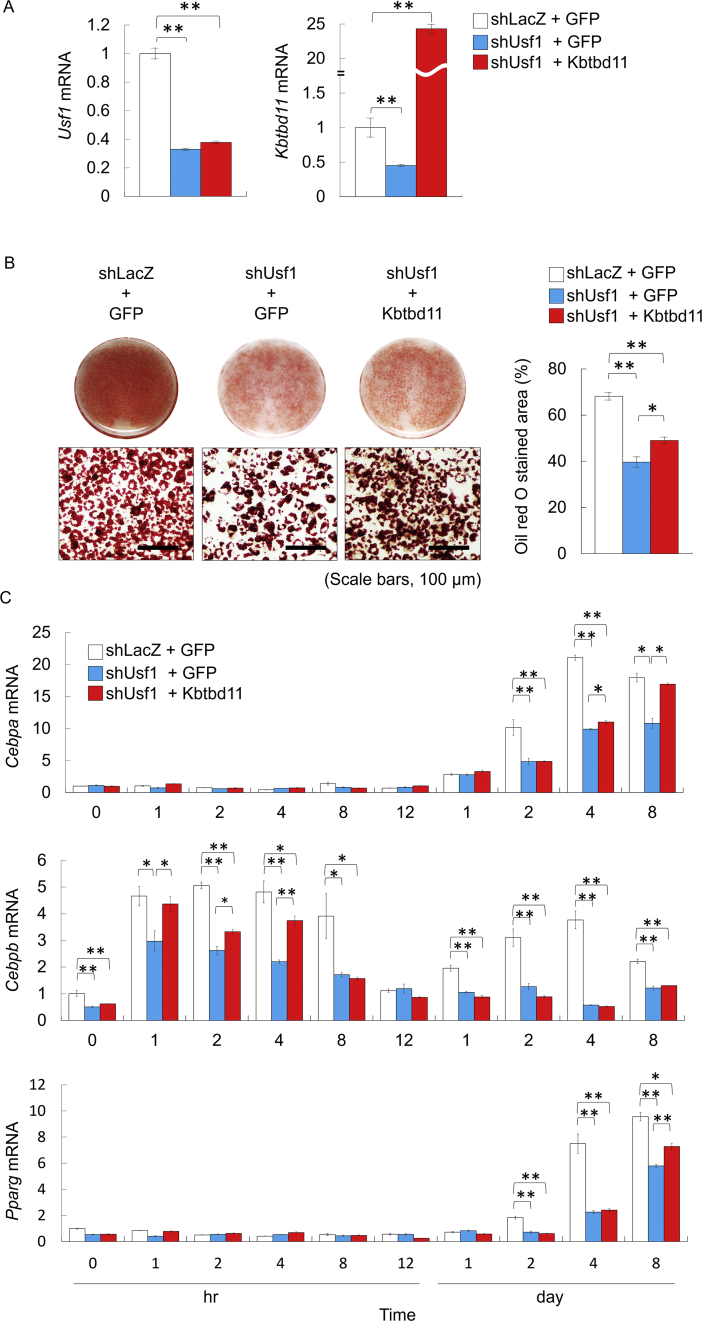
The effects of *Kbtbd11* overexpression on *USF1* knockdown 3T3-L1 cells. The 3T3-L1 cells were infected with mixture adenoviral vectors (at an MOI of 30) for expressing shLacZ, GFP, shUsf1, and *Kbtbd11* at day −2 before inducing differentiation and were then incubated for 8 days. (A) The expressions of *Usf1* and *Kbtbd1*1 mRNA in *Usf1* knockdown 3T3-L1 cells at day 8. *n* = 3 per group, *p < 0.05, **p < 0.01 vs. shLacZ+GFP. (B) Triglyceride accumulation in 3T3-L1 cells on day 8 was visualized using Oil Red O staining (scale bar = 100 μm) (left), quantification of Oil Red O stained area (right). (C) The mRNA levels in 3T3-L1 cells expressing each adenovirus mixture (shLacZ+GFP, shUsf1+GFP, and shUsf1 and Kbtbd11) at various time points after inducing differentiation. *n* = 3 per group, *p < 0.05, **p < 0.01 vs. shLacZ+GFP. Relative mRNA levels for adipogenic markers (*C/ebpa*, *C/ebpb*, and *Pparg*) were estimated after normalization with *Rplp0*. *n* = 3 per group, *p < 0.05, **p < 0.01 vs. shLacZ+GFP.

To investigate the role of *Kbtbd11* in adipocyte differentiation, we examined the effects of *Kbtbd11* in *Usf1* knockdown 3T3-L1 cells. The adenoviral coinfection of *Usf1* shRNA and GFP (shUsf1+GFP) constructs reduced *Usf1* mRNA expression levels by up to 70% compared with the coinfection of shLacZ and GFP (shLacZ+GFP) control (Fig. 8A). On day 8 after inducing differentiation, shLacZ+GFP control cells showed lipid droplet abundance (observed with ORO staining) (Fig. 8B) and increases in the expressions of *C/ebpa*, *C/ebpb*, and *Pparg* (Fig. 8C). Consistent with the reduction in *Usf1* expression in the coinfected shUsf1+GFP 3T3-L1 cells, *Kbtbd11* expression decreased by 50 % compared with that in the shLacZ+GFP control. On day 8 after inducing differentiation, shUsf1+GFP 3T3-L1 cells exhibited a marked reduction in lipid accumulation (Fig. 8B) and significant decreased in adipocyte differentiation marker levels (*C/ebpa*, *C/ebpb*, and *Pparg*) (Fig. 8C).

In *Kbtbd11*-overexpressing *Usf1* knockdown 3T3-L1 cells, the *Kbtbd11* mRNA expression increased by 24-folds (Fig. 8A). On day 8 after inducing differentiation, *Kbtbd11*-overexpressing *Usf1* knockdown cells exhibited a significant reduction in both lipid accumulation (Fig. 8B) and adipocyte differentiation marker levels (*C/ebpa*, *C/ebpb*, and *Pparg*) (Fig. 8C) compared with those in the shLacZ+GFP control. However, in *Kbtbd11*-overexpressing *Usf1* knockdown 3T3-L1 cells, *C/ebpb* expression during the early stage of adipogenesis increased significantly compared with *C/ebpb* expression in shUsf1+GFP 3T3-L1 cells (Fig. 8C). Compared with that in shUsf1+GFP 3T3-L1 cells, following rescued *C/ebpb* expression in shUsf1+Kbtbd11 3T3-L1 cells, *C/ebpa* and *Pparg* expressions in the late stage of adipogensis were fairly rescued by *Kbtbd11* overexpression (Fig. 8C).

### 3.8 Depression of adipogenic and lipogenic genes in *Usf1* knockdown of mature 3T3-L1 adipocytes recovered by *Kbtbd11* overexpression

**Fig. 9:**
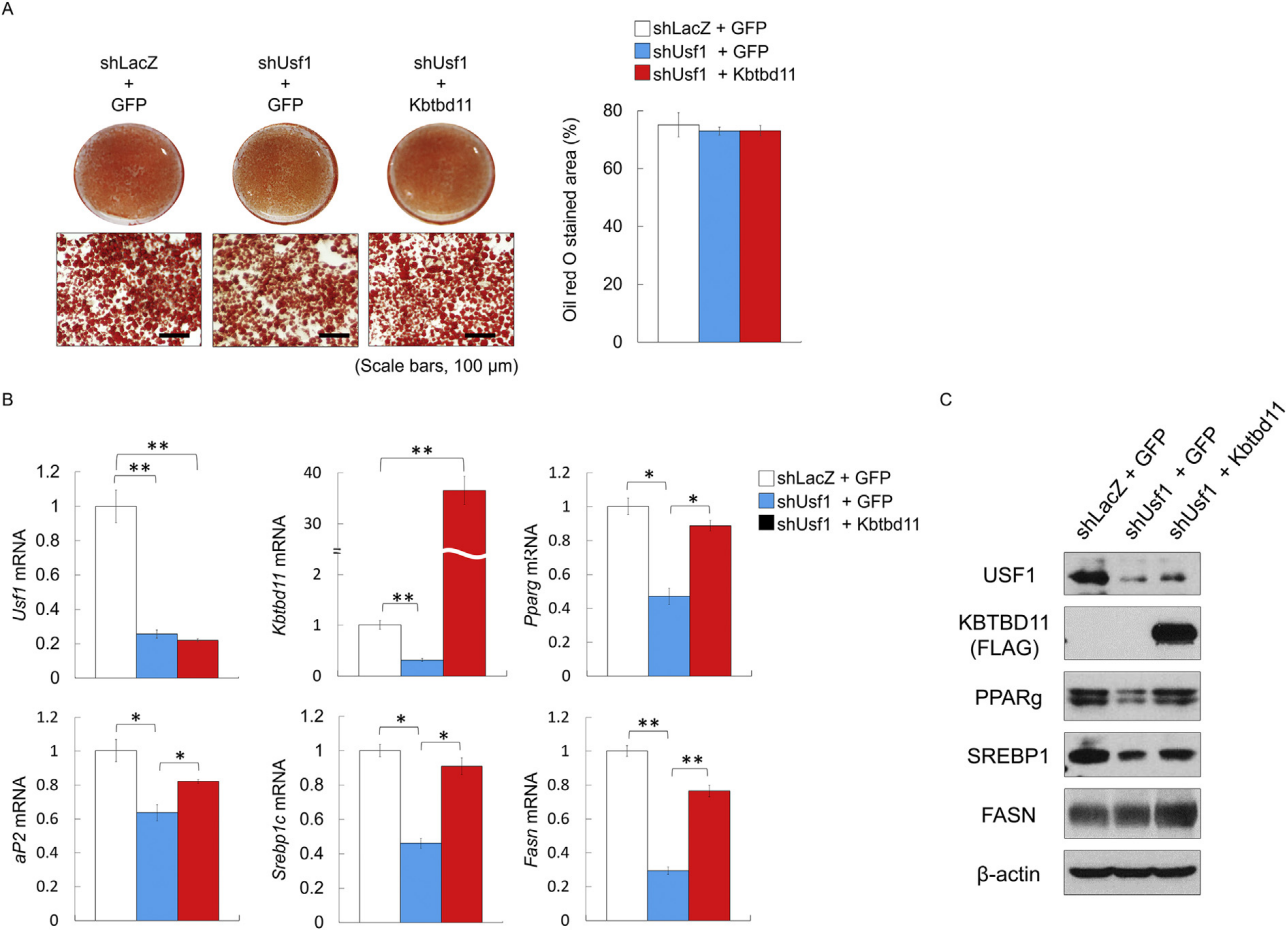
Effects of *Kbtbd11* overexpression on *Usf1* knockdown mature 3T3-L1 adipocytes. Mature 3T3-L1 cells were infected with adenoviral vectors (shLacZ, shUsf1, GFP, and *Kbtbd11*) at an MOI of 30. The differentiated mature 3T3-L1 adipocytes at day 8 after induction were used. (A) Triglyceride accumulation in mature 3T3-L1 adipocytes 48 h after each adenoviral mixture infection (shLacZ+GFP, shUsf1+GFP, and shUsf1and Kbtbd11) visualized using Oil Red O staining (scale bar = 100 μm) (left), quantification of Oil Red O stained area (right); (B) Total RNA was harvested 48 h after adenoviral infection, and expression levels of *Usf1*, *Kbtbd11*, and adipogenic (Pparg and aP2), and lipogenic (Srebp1c and Fasn) genes were measured using qPCR; *n* = 3 per group, *p < 0.05, **p < 0.01 vs. shLacZ+GFP. (C) Western blotting showing USF1, KBTBD11, PPARg, SREBP, FASN, and β-actin protein levels.

To examine the role of *Kbtbd11* in mature adipocytes, we investigated the effects of *Kbtbd11* in *Usf1* knockdown mature 3T3-L1 adipocytes. On day 8 after inducing differentiation, mature 3T3-L1 adipocytes were coinfected with adenoviral vectors expressing shLacZ and GFP control, shUsf1 and GFP, or shUsf1 and *Kbtbd11*. Compared with shLacZ and GFP control, coinfection of shUsf1 and GFP decreased *Usf1* mRNA expression levels (by up to approximately 80%) (Fig. 9B) as well as USF1 protein levels (Fig. 9C). Consistent with the reduction in *Usf1* expression under coinfection of shUsf1+GFP 3T3-L1 cells, *Kbtbd11* expression decreased by 70 % compared with *Kbtbd11* expression in shLacZ+GFP cells. In *Kbtbd11*-overexpressing *Usf1* knockdown 3T3-L1 cells, *Usf1* mRNA expression decreased by 80 % and *Kbtbd11* mRNA increased by 36-folds compared with that in shLacZ+GFP cells (Fig. 6B). Endogenous KBTBD11 protein levels could not be detected using a commercial antibody. Because the *Kbtbd11*-overexpressing adenovirus vector was fused with FLAG tag, only overexpression of KBTBD11 was detected by the anti-FLAG antibody (Fig. 9C). The lipid droplet abundance in mature 3T3-L1 adipocytes did not vary among shLacZ+GFP control, shUsf1+GFP cells, and shUsf1+*Kbtbd11* cells (observed using ORO staining) (Fig. 9A). In addition, lipid droplet abundance was not different in mature 3T3-L1 adipocyte cells under shUsf1+GFP and shLacZ+GFP conditions (observed using ORO staining) (Fig. 9A). However, adipogenic (*Pparg* and *aP2*) and lipogenic (*Srebp1c* and *Fasn*) genes significantly decreased in the shUsf1+GFP mature 3T3-L1 adipocyte cells (Fig. 9B). PPARg and SREBP1 protein levels were consistent with *Pparg* and *Srebp1c* mRNA levels in the shUsf1+GFP cells (Fig. 9C). Similarly, lipid droplet abundance was not different between in mature 3T3-L1 adipocytes under shUsf1+Kbtbd11 and shLacZ+GFP conditions (observed using ORO staining) (Fig. 9A), although adipogenic (*Pparg* and *aP2*) and lipogenic (*Srebp1c* and *Fasn*) genes increased significantly and nearly recovered in the shLacZ+GFP control (Fig. 9B). PPARg and SREBP1 protein levels were increased in the shUsf1+Kbtbd11 cells compared with those in the shUsf1+GFP cells (Fig. 9C). FASN protein levels were not different between the shUsf1+GFP and shLacZ+GFP cells; however, FASN protein levels were higher in shUsf1+Kbtbd11 cells (Fig. 9C).

## 4 Discussion

We have previously reported that *Kbtbd11* mRNA expression increases in a time-dependent manner during 3T3-L1 differentiation and increases significantly in eWAT under feeding status and obesity [2]. Moreover, we reported that *Kbtbd11* knockdown inhibits mitotic clonal expansion (MCE), and the resulting *Kbtbd11* attenuates 3T3-L1 adipocyte differentiation. Unlike *Kbtbd11* knockdown in 3T3-L1 cells, *Kbtbd11* overexpression promotes MCE, thus leading to the expressions of *C/ebpa* and *Pparg.* These results suggest that *Kbtbd11* expression regulation plays an important role in adipogenesis. Therefore, we hypothesized that the elevated expression of *Kbtbd11* in eWAT in obese mice during 3T3-L1 differentiation could be regulated by transcription factors, including adipogenic and lipogenic transcription factors.

To examine the regulation of *Kbtbd11* expression in 3T3-L1 cell differentiation, we investigated *Kbtbd11* transcriptional control mechanisms. We used LASAGNA-Search 2.0 for analyzing the candidate transcription factor binding sites in *Kbtbd11* promoter and identified the potential binding sites of lipogenic and adipogenic transcription factors, SREBP1, LXR, USF1 C/EBPa, PPARg, and HNF4a. Subsequently, we investigated whether the transcription factors activate the *Kbtbd11* promoter and found that USF1 was bound to the Ebox motif in the *Kbtbd11* promoter and it enhanced the activation of *Kbtbd11* transcription significantly. In fasting–refeeding experiments, *Usf1* mRNA expression was increased in a time-dependent manner during chow diet refeeding, which is consistent with the *Kbtbd11* mRNA expression. In addition, the attenuation of *Usf1* mRNA after HF diet feeding was also consistent with that of *Kbtbd11* mRNA (Fig. 5). Furthermore, we evaluated plasma insulin and glucose levels in fasting–refeeding state. The HF diet refeeding after caloric restriction reduced insulin sensitivity and increased circulating plasma insulin levels [12]. Plasma insulin levels were significantly increased during HF refeeding compared with those during chow diet refeeding. Plasma glucose levels were significantly decreased after HF refeeding, accompanied with the increase in plasma insulin levels (Fig. 6). Because glucose increases *USF1* expression [11], the reduced plasma glucose levels after HF refeeding might downregulate USF1. Indeed, *Usf1* mRNA was decreased after HF refeeding compared with that during chow diet refeeding (Fig. 5), resulting in the reduced expression of *Kbtbd11*.

USF1 is a basic helix–loop–helix leucine zipper transcription factor, which recognizes the Ebox motif (CACGTG) [13]. USF1 is ubiquitously expressed, and it regulates several proteins involved in lipid metabolism, including C/EBPa [14], fatty acid synthase (FASN) [13], and acetyl-CoA carboxylase-α [15]. In addition, the genetic variants of *USF1* are associated with familial combined hyperlipidemia [16, 17], increased risk of cardiovascular diseases [17, 18], risk of type 2 diabetes [19, 20], metabolic syndrome traits [21, 22], and increased levels of obesity [23, 24, 25]. Because *Kbtbd11* is a USF1 target gene and is altered by nutritional regulation (Fig. 2) during adipocyte differentiation [2], it plays an important role in lipid metabolism. However, *Kbtbd11* upregulation might not play a crucial role in the modulation of mature adipocyte function(s), except after starvation or during energy storage in adipose tissue (Fig. 7).

In addition, we revealed that the effect of *Usf1* knockdown in 3T3-L1 cell differentiation is rescued through *Kbtbd11* overexpression. *Usf1* knockdown inhibits differentiation into 3T3-L1 adipocytes because USF1, a crucial master regulator of adipocyte differentiation, regulates the transcription of *C/ebpa* and *Fasn* by binding to the Ebox region of the promoters [26]. However, on day 4 after inducing differentiation, adipocyte differentiation marker (*C/ebpa*, *Pparg*, *Fasn*, and *aP2*) levels increased significantly *Kbtbd11*-overexpressing *Usf1* knockdown cells compared with those in *Usf1* knockdown cells (Fig. 8C). These results suggest that the differentiation of *Usf1* knockdown 3T3-L1 cells could be partially rescued by *Kbtbd11* overexpression. Furthermore, mature 3T3-L1 adipocyte cells under shUsf1+Kbtbd11 condition showed that lipid droplet abundance was not different when compared with the abundance in the shLacZ+GFP control (observed with ORO staining) (Fig. 9A). However, adipogenic (*Pparg* and *aP2*) and lipogenic (*Srebp1c* and *Fasn*) genes significantly increased and nearly recovered in the shLacZ+GFP control (Fig. 9B), suggesting that maintenance of *Kbtbd11* expression could be essential during adipocyte differentiation and/or adipose tissue homeostasis.

Moreover, *Kbtbd11* overexpression did not affect the expressions of adipocyte differentiation markers in 3T3-L1 cells. This result was consistent with *Kbtbd11* knockdown in mature 3T3-L1 cells, wherein *Kbtbd11* knockdown in mature 3T3-L1 cells was not different from the control adipocytes [2]. These results suggest that *Kbtbd11* did not affect the already matured adipocytes and/or adipocytes that have reached the lipogenesis threshold. Conversely, *Usf1* knockdown in mature 3T3-L1 cells downregulated adipogenic and lipogenic genes (*Pparg*, *aP2*, *Srebp1c*, and *Fasn*) (Fig. 9). In such conditions (wherein the lipogenesis threshold is not reached), *Kbtbd11* could play a role in the maintenance of adipocyte homeostasis. However, the molecular mechanism(s) of *Kbtbd11* in adipocyte homeostasis and the physiological functions remain to be fully elucidated.

A recent study has reported the methylation and gene expression profiling of B-cell acute lymphoblastic leukemia (ALL) using next-generation sequencing and showed that *KBTBD11* differed in methylation and expression levels between two subtypes of patients (TCF3-HLF and TCF3-PBX1) based on the presence of chromosomal translocations [27]. *KBTBD11* was overexpressed in TCF3-HLF compared with that in TCF3-PBX1 patients, and the CpG sites in the exons of *KBTBD11* were hypomethylated before remission in TCF3-HLF patients. Following remission, *KBTBD11* methylation increased and gene expression was downregulated. The results suggested that *KBTBD11* hypomethylation, expression levels, and/or transcriptional regulation are associated with the regulation of B-cell proliferation and differentiation in B-cell ALL [28].

In addition, the oncogenic transcription factor MYC bound to a MYC response element in *KBTBD11* intron1 and regulated *KBTBD11* expression [4, 29]. Furthermore, rs11777210, a variant allele of *KBTBD11* in the MYC response element in *KBTBD11* intron1, is significantly associated with cell susceptibility to colorectal cancer. *KBTBD11* is reported to be downregulated in tumor tissues, and *KBTBD11* knockdown promoted cell proliferation and inhibited cell apoptosis [4], suggesting that regulation of *KBTBD11* expression and transcript levels of *KBTBD11* play important roles in tumorigenesis and function.

Overall, alterations in *KBTBD11* expression levels, methylation, and transcriptional regulation using a variant of MYC and a subtype of TCF3-HLF could be an important proliferation and differentiation function in various cell types. Considering that *Kbtbd11* is a target USF1 gene that regulates proliferation and differentiation in adipocytes, genetic variation in USF1 and varying *Kbtbd11* expression levels could be associated with obesity.

In conclusion, we revealed that USF1, an important regulator of lipid metabolism, is involved in the transcriptional regulation of *Kbtbd11* and found that the effect of *Usf1* knockdown is rescued through *Kbtbd11* overexpression. However, further research is required to clarify *Kbtbd11* methylation and expression differences between normal and obese individuals and elucidate the physiological function(s) of *KBTBD11*. KBTBD11 can be applied to control the progression and differentiation, including the development, of various cell types, such as adipocytes and cancer cells.

## Declarations

### Author contribution statement

Kazuhisa Watanabe: Conceived and designed the experiments; Performed the experiments; Analyzed and interpreted the data; Contributed reagents, materials, analysis tools or data; Wrote the paper.

Kazuha Yokota, Ken Yoshida, Ayumi Matsumoto, Sadahiko Iwamoto: Contributed reagents, materials, analysis tools or data.

### Funding statement

This work was supported by grant-in-aid (15K19523, 17K16153 and 17K09864) from Japan Society for the Promotion of Science (JSPS; MEXT program of supporting for the strategic research bases at private universities (2013–2017); Jichi Medical University young investigator award.

### Competing interest statement

The authors declare no conflict of interest.

### Additional information

No additional information is available for this paper.

## Figures and Tables

**Supplementary material for reference:**